# Comments on “Right Ventricular Hydatid Cyst Before and After Rupture”

**DOI:** 10.1002/ccr3.72923

**Published:** 2026-06-10

**Authors:** Hakan Baltacı, Zihni M. Duman

**Affiliations:** ^1^ Department of Cardiovascular Surgery Elazıg City Hospital Elazıg Turkey

**Keywords:** albendazole, anaphylactic shock, cardiac hydatid cyst, hydatid cyst rupture, right ventricular mass

## Abstract

Early surgical excision should be considered the primary treatment modality for cardiac hydatid cyst, and that medical therapy should be meticulously integrated into the preoperative planning process.

We read with interest the article “Right Ventricular Hydatid Cyst Before and After Rupture” by Rashid et al. [1], recently published in *Clinical Case Reports*. We congratulate the authors. We would like to discuss some points about the timing of surgery.

In the case, the authors describe a 21‐year‐old male patient who presented with symptoms of chest pain, dyspnea and palpitations. Imaging studies demonstrated a 4.5 × 3.5 cm thin‐walled cardiac hydatid cyst (CHC) with faint peripheral calcification in the right ventricular apex. Albendazole therapy was initiated, and elective surgical intervention was planned. During the two‐month follow‐up period, anaphylactic shock developed following rupture of the hydatid cyst.

CHC are constantly at risk of rupture due to continuous myocardial contraction and pressure changes and rupture is considered the most fatal complication [2]. Surgical treatment can be performed with low surgical risk and is the primary treatment method because the risk of rupture is high regardless of symptoms [3, 4].

Preoperative albendazole treatment appears reasonable for reducing cyst viability and lowering the risk of recurrence. On the other hand, Usluer et al. [5] demonstrated that preoperative albendazole treatment may cause thinning and fragmentation of the cyst's cuticular membrane, potentially facilitating intraoperative or spontaneous rupture in pulmonary hydatid cysts. In spite of some reports in the literature describing a reduction in the size of CHC with medical treatment, such favorable responses are rare and have been observed in only a few cases requiring long‐term follow‐up [6, 7, 8]. In CHC, medical therapy should be used as adjuvant treatment to surgery. On the contrary, if the CHC carries a risk of rupture or calcification, prolonged preoperative medical treatment is not recommended [3].

The absence of guidelines on the optimal treatment of CHC, especially the duration of preoperative medical therapy, has resulted in significant variability in clinical practice.

Meticulous use of serial echocardiography and cardiac magnetic resonance imaging in the preoperative period may facilitate optimal surgical timing before rupture occurs, especially in patients with high‐risk anatomical features. Based on this case, the current literature, and our clinical experience, we suggest that early surgical excision should be considered the primary treatment modality for CHC, and that medical therapy should be meticulously integrated into the preoperative planning process. This approach has been adopted as standard practice in our institution, and early elective surgical treatment is routinely performed in patients with CHC [9].

We would like to kindly ask the authors for their comments regarding the delay of surgery in a thin‐walled CHC with faint peripheral calcification which is known to carry a high risk of rupture:

1. Was there a specific clinical reason for postponing surgery for 2 months?
2. Were serial imaging studies performed during the follow‐up period to assess changes in cyst wall morphology?
3. In light of this experience, what is the authors' current perspective on the safe upper limit for the duration of preoperative albendazole therapy in cardiac hydatid cysts?

We would appreciate the authors' valuable comments on these points.

## Author Contributions

**Hakan Baltacı:** writing – review and editing, writing – original draft, supervision. **Zihni M. Duman:** writing – review and editing, writing – original draft, supervision.

## Funding

The authors have nothing to report.

## Ethics Statement

The authors have nothing to report.

## Conflicts of Interest

The authors declare no conflicts of interest.

## Data Availability

Data sharing not applicable to this article as no datasets were generated or analysed during the current study.
